# Influence of Mo on Ni-15Cr Cladding Layers via Plasma Transferred Arc

**DOI:** 10.3390/ma15238381

**Published:** 2022-11-24

**Authors:** Peiran Shi, Hang Yin, Yichen Zhou, Guodong Zhang

**Affiliations:** School of Power and Mechanical Engineering, Wuhan University, Wuhan 430072, China

**Keywords:** cladding layer, plasma transferred arc, microstructure, micro hardness, corrosion resistance

## Abstract

The composite Ni-Cr-Mo covering layers with excellent corrosion and wear resistance are deposited by plasma transferred arc (PTA), which can improve the service life of ships and solve the corrosion and wear problems of mechanized boats. The effects of Mo on the microstructure, hardness and corrosion resistance of covering layers were analyzed by OM, SEM, XRD, EDS, a micro hardness test, a friction test and a corrosion-resistance test. The results show that the structure of covering layers change and the austenite precipitates become granular with an increase of Mo content. In addition, the corrosion and wear resistance of covering layers are improved and the sample with 5% content of Mo has the best wear and corrosion resistance.

## 1. Introduction

As an important part of the ecosystem, the ocean provides necessary and precious resources for human survival and development. However, Cl^−^ in the ocean is highly corrosive and easily destroys oxide films of steel components to make materials fail [1,2,3]. Therefore, effective measures must be taken for metal components in marine service. With low cost, high operability and simple maintenance, coating technology is widely used for the protection of low-carbon steel hulls. However, the thermal stress makes coating layers shrink, expand and peel from ships [4,5]. In addition, some coatings with poor mechanical properties, such as hardness and wear resistance, are worn away during the work and cannot protect hulls and parts of mechanized boats from corrosion. As a result, better covering layers with excellent corrosion and wear resistance must be prepared to protect mild steel hulls.

PTA is one of the most used technologies for low carbon steel owning to high production efficiency, simple equipment, high performance and low dilution rate of covering layers [6,7,8,9]. Nowadays, coating powders with excellent wear and corrosion resistance, such as Fe-based, Ni-based and Co-based powders, are widely used in the protection of hulls and parts of mechanized boats [10,11,12,13,14,15]. Co-based alloys with high cost are mostly used for expensive industrial components. Ni-based alloys have not only excellent properties but also lower prices than cobalt-based alloys [16,17,18,19]. The existing Ni60 is used to prepare Ni-based plasma covering layers. However, the covering layers have poor surface quality with pores. A single type of alloy powder cannot meet service requirements in an extremely harsh service environment. The wear and corrosion resistance of alloys is enhanced by adding hard ceramic phases [20,21,22,23]. Ni-Cr-Mo covering layers have the advantages of Cr-based, Mo-based and Ni-based alloys [24]. However, the existing ratios of Ni, Cr, and Mo powder are not appropriate for the preparation of plasma covering layers of low-carbon steel, so it is necessary to explore the appropriate powder composition ratio. 

In this paper, the Ni-Cr-Mo composite covering layers were prepared by PTA, which can be applied to hulls and parts of mechanized boats. It can improve the service life of ships and solve the corrosion and wear problems of mechanized boats, which has a practical application prospect and profound development significance.

## 2. Materials and Methods

### 2.1. Materials

The base material is Q235 steel, which is widely used in mechanized boats. The material of the powder is composed of pure Ni, Cr and Mo powder. The particle sizes are 50–100 μm. The chemical composition of Q235 steel and powder composition of covering layers is shown in Table 1 and Table 2.

### 2.2. Preparation of the Cladding Layer

The Q235 steel plate with the size of 100 × 100 × 10 mm^3^ was polished by a grinder and polished further with 15 μm sandpaper until it became smooth. Then it was placed in alcohol solution for ultrasonic cleaning for 30 min. After that, the steel plate was put in a dryer at 65 °C for 1 h and placed on the heat-dissipation metal of the plasma cladding worktable. High-energy plasma arc powder-surfacing equipment (PTA-BX-400A, Shanghai Benxi Electromechanical Technology, Shanghai, China) was used for this experiment. The parameters of the cladding process are shown in Table 3.

### 2.3. Test Method 

Covering layers were cut to the size of 10 × 10 × 10 mm^3^ by a cutter. Then, the covering layers were polished and cleaned. Smooth and clean samples with the size of 10 × 10 × 10 mm were obtained and then examined by optical microscope (OM, LAB-1, CARL ZEISS AG, Oberkochen, Germany), X-ray diffractometer (XRD, smart lab, Rigaku, Tokyo, Japan), energy spectrum analyser (EDS, Aztec Energy X-Max20, Hitachi Limited, Tokyo, Japan) and Field Emission Scanning Electron Microscope (SEM, MIRA 3 LMH, TESCAN, Brno, Czech) for their microstructures. 

The hardness of the covering layers was measured with a Vickers hardness tester (HXS-1000A, Shanghai Shuangxu Electronics, Shanghai, China) under the condition of 3 N test force and 10 s press time. A friction tester (MS-T3000, Lanzhou Huahui Instrument Technology, Lanzhou, China) was used for friction tests at room temperature with a load of 2 N and a speed of 200 r min^−1^. The size of samples was 15 × 15 × 5 mm^3^ and the material of grinding balls was Si_3_N_4_. The corrosion resistance of the covering layers was explored with an electrochemical workstation (CS310H, Wuhan Corrtest Instruments, Wuhan, China).

## 3. Results and Discussion

### 3.1. Microstructure and Composition Analysis

**Figure 1:**
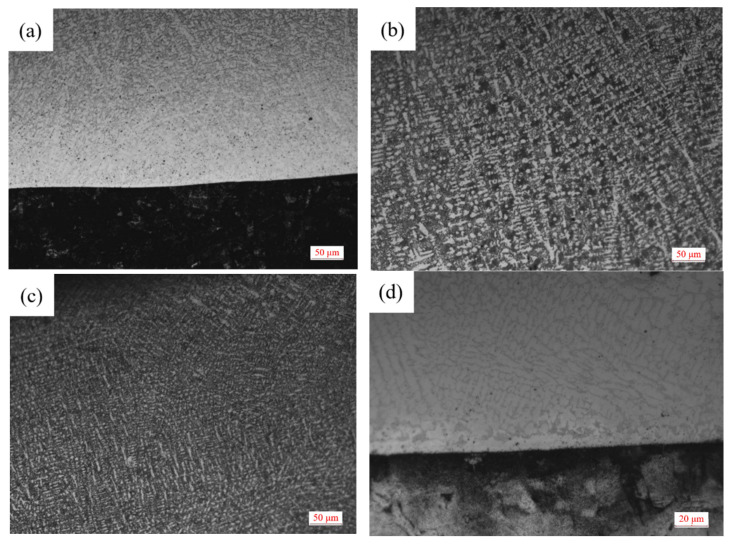
Microstructure of cladding layer of Sample 1. (**a**) Bottom image. (**b**) Middle image. (**c**) Top image. (**d**) Bottom high magnification image.

The microstructure of Sample 1 covering layer under OM is shown in Figure 1. It can be seen from Figure 1a,d that the Q235 steel plate has been completely corroded and the structure can not be distinguished. Moreover, there is a bright white fusion line between the matrix and the bottom of the covering layer which shows that the steel and the layer bond well. Due to the connection between the top of the molten pool and the air, the heat is lost from the solid-gas interface during the solidification of the molten pool. Compared with the middle of the covering layer, the cooling speed of the top zone is faster and the dendrites formed in the top Zone A are smaller. 

**Figure 2:**
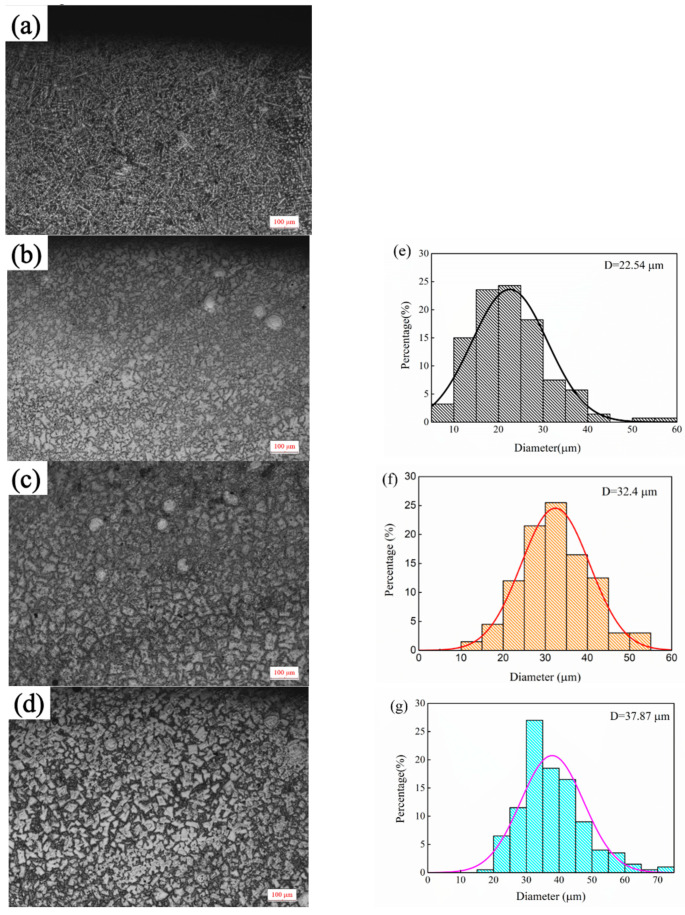
Micromorphology of top covering layers with different contents of Mo. (**a**) 0 wt.% Mo (**b**) 5 wt.%. Mo (**c**) 10 wt.% Mo. (**d**) 15 wt.% Mo; Grain size distributions. (**e**) Sample 2. (**f**) Sample 3. (**g**) Sample 4.

The microstructure of top covering layers with different contents of Mo is presented in Figure 2. The top microstructure of Sample 1 without Mo is a typical dendritic structure. In Figure 2a, the black part is the zone of the matrix phase containing a lot of Fe and Ni, and the white acicular and punctiform structure is caused by the precipitation of Cr. In Figure 2b, the structure of the austenite-precipitated phase changes from dots and needles to granules. In Figure 2c,d, the distribution-of-austenite phase becomes more even and the grains become coarser. The main reason for the phenomena in Figure 1a,b is that the Mo powder cannot melt completely with the increase of Mo content in the molten pool. Since the melting temperature of Mo is much higher than that of Ni, Cr and Fe, Mo is the first to precipitate as nucleation particles during the solidification of the molten pool, which increases the rate of heterogeneous nucleation and promotes the refinement of the microstructure. In addition, a fraction of C and Si in the matrix enters the molten pool to form carbides and silicides with Mo due to the strong affinity with Mo, which further refines grains. The main reason for the phenomena in Figure 1c,d is that the heat released during the solidification of Mo is greater and the cooling time of the covering layer increases with the further increase of Mo content, which promotes grain growth and coarsening.

**Figure 3:**
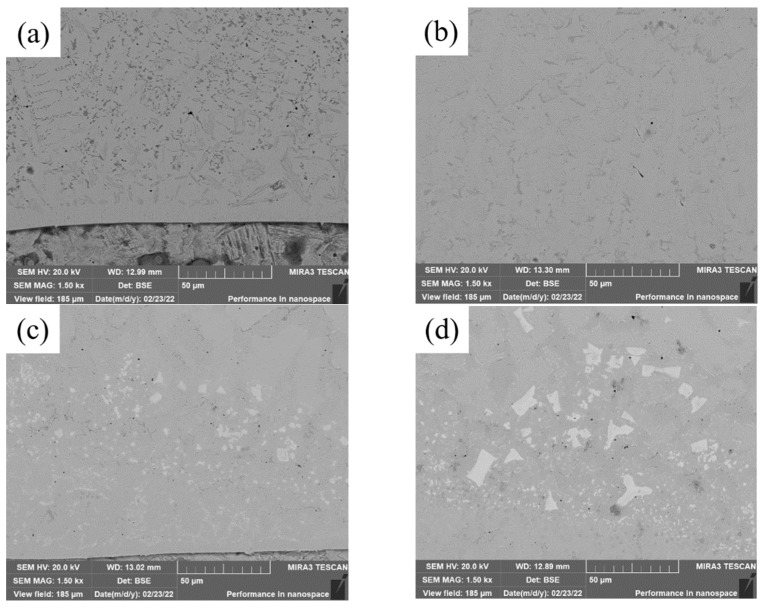
SEM images of the bottom of cladding layers with different contents of Mo. (**a**) 0 wt.% Mo. (**b**) 5 wt.% Mo. (**c**) 10 wt.% Mo. (**d**) 15 wt.% Mo.

SEM images of the bottom of covering layers with different contents of Mo are visualized in Figure 3. With the increase of Mo content, the content of acicular and punctiform precipitates decreases and the content of fishbone-like precipitates increases. In addition, small and black pores are found in the four covering layers and the numbers of pores in Figure 3b,c is significantly less than that in Figure 3a, indicating that the addition of Mo content is conducive to the reduction of the pores of covering layers.

**Figure 4:**
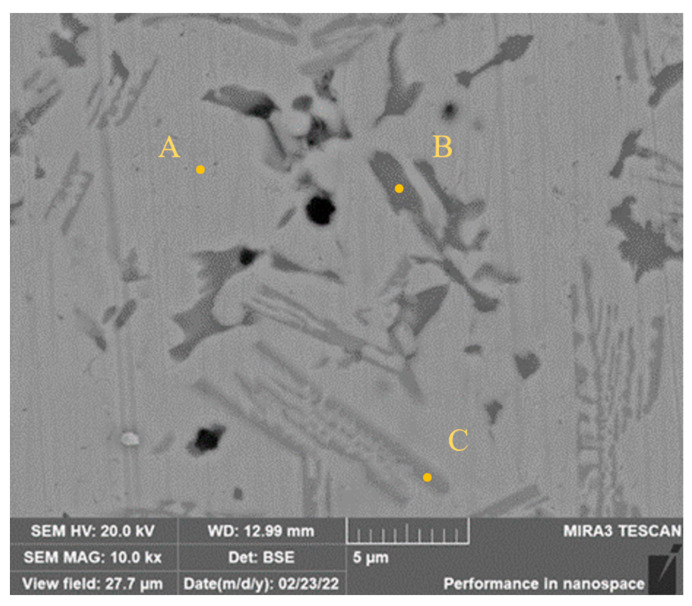
High magnification SEM image of holes.

The high-magnification SEM image of pores in Sample 1 is shown in Figure 4. Most of the pores distribute in the zone of dark austenite, and there are few pores in the zone of the matrix. Combined with EDS analysis presented in Table 4, the Ni-rich matrix phase with 60.23 wt.% content of Ni can be found in Zone A. The dark phase in Zone B is the Cr-rich austenitic phase with 59.43 wt.% content of Cr and most of the pores are in this phase. The content of Cr in Zone C is 22.62 wt.%, which is higher than that of the matrix phase, and the contents of Fe in three zones are similar. It is speculated therefore that the formation of pores is related to the segregation of Cr and the degree of segregation reduces with the increase of Mo content.

**Figure 5:**
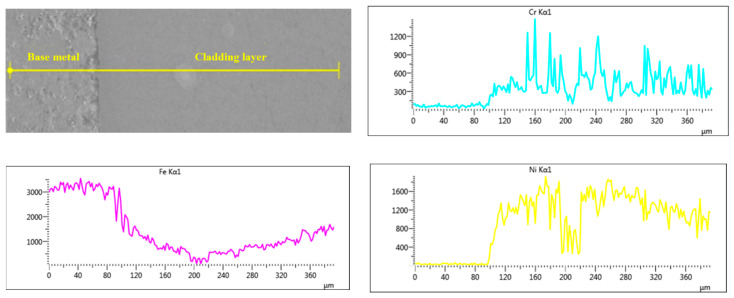

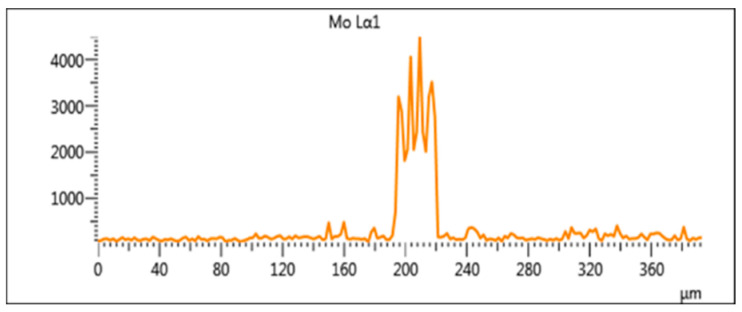
EDS line scan element distribution near the fusion line.

The EDS scan element distribution of Sample 2 near the fusion line is presented in Figure 5. The contents of Cr, Ni and Mo in the base metal are almost 0 and the concentrations of the three elements increase sharply from the fusion line to the covering layer. It can be speculated that Ni, Cr and Mo elements in the covering layer diffuse with difficulty to the base metal and the dilution rate of the base metal is low, which ensures not only the good metallurgical combination between the base metal and the covering layer but also the high purity of the covering layers. In Figure 5, the concentration of Fe in the covering layer remains almost unchanged with a certain concentration, which indicates that the base metal melts slightly during the formation of the molten pool and some of Fe element diffuses into the covering layer. In addition, the peak value of Mo is particularly high near 200 μm, which indicates that Mo is deposited at the bottom of the covering layer as Mo particles.

**Figure 6:**
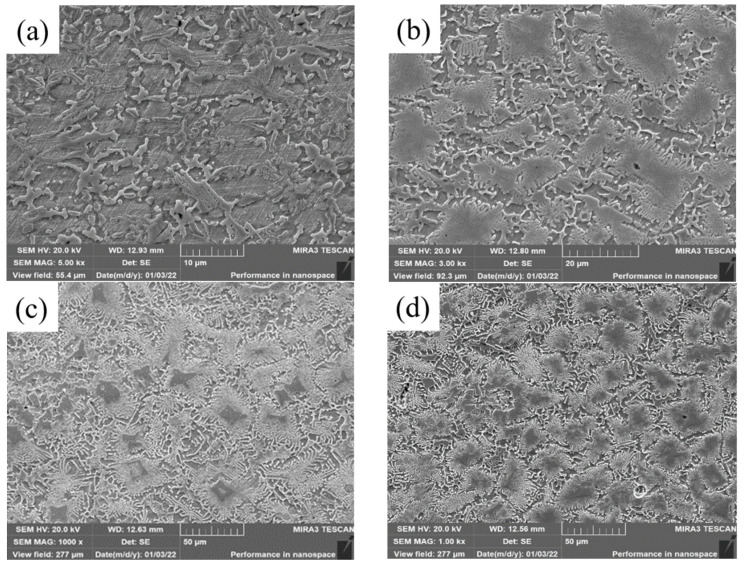
SEM images of middle of cladding layers with different Mo contents. (**a**) 0 wt.% Mo. (**b**) 5 wt.% Mo. (**c**) 10 wt.% Mo. (**d**) 15 wt.% Mo.

In Figure 6, the distribution of the precipitated phase changes with the increase of Mo content. The structure of the sample 1 precipitated phase is columnar, irregular strip or puncta. With the addition of Mo, there is agglomeration of the precipitated phase in samples 2, 3 and 4.

**Figure 7:**
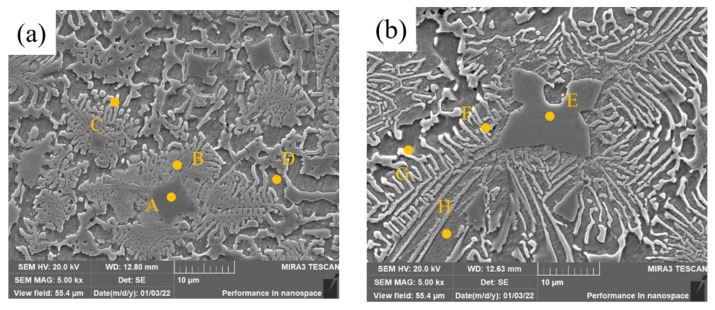
High-magnification SEM images of cladding layers with different contents of Mo. (**a**) 5 wt.% Mo. (**b**) 10 wt.% Mo.

High-magnification SEM images of middle covering layers of samples 2 and 3 are shown in Figure 7. The precipitated phase mainly consists of three parts: the dark blocky zone, the light strip Zone And the bright white strip zone. The bright white zone is mostly at the edge of the dark strip zone. Eight small zones were selected for EDS microzone element tests, and the results are shown in Table 5.

Precipitates in Zone A are rich in Cr and Mo and poor in Fe and Ni. C element can be found in this zone, and the reason is that C in the matrix enters the molten pool and forms carbides with Cr and Mo. In the process of the molten pool solidification, the carbides are precipitated first as non-uniform nucleation particles owing to the higher freezing point. As well, Cr, Mo and austenitic with lots of Cr and Mo crystallize and grow on the carbide particles, making the contents of Mo and Cr relatively high in Zone A. Compared with Zone A, the contents of Ni and Fe in Zone B are higher, while the contents of Cr and Mo are significantly lower. Due to the low freezing point of the Ni and Fe solid solution, the solid solution nucleates, grows on the crystallized austenitic phase (near Zone B) and spreads in strips to divergent directions during the solidification. Therefore, the content of Ni at the end of the strips, such as in Zone C, is 80.21 wt.% and the content of Cr and Mo are 6.71 wt.% and 3.80 wt.%, which are the lowest. The matrix was corroded by the metallographic etching liquid in Zone D, where the contents of Ni and Fe are high and there is no Mo.

The content of Mo in Zone E is higher than that in Zone A, and the black blocky zone, which is caused by the incomplete melting of Mo particles with the increase of Mo content, is larger. Compared with zones B and C, the contents of Mo and Cr in zones F and G increase because the heat releases more with the content of Mo, owing to the higher freezing point of Mo, which increases the cooling time of the covering layers and promotes the diffusion and solidification of Mo. The content of Mo element in Zone H is 6.25 wt.% while there is no Mo in Zone D, which also proves that Mo atoms diffuse better in Sample 3 than in Sample 2.

**Figure 8:**
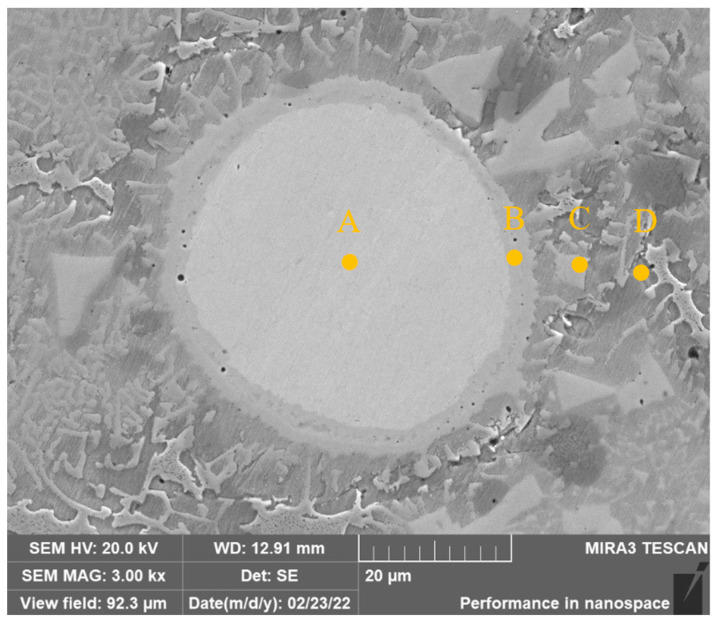
SEM image of unmelted Mo particles of Sample 4.

Figure 8 shows the high-magnification SEM image of the unmelted Mo particles in the covering layer of Sample 4. The EDS element analysis in Table 6 shows that there is only Mo element in Zone A which indicates Ni, Cr and Fe atoms have not diffused into Mo particles. Zone B is the melting zone of Mo where Mo particles dissolve and diffuse to form solid solutions or compounds with Ni, Cr and Fe. The highest Ni content of 23.59 wt.% can be found in Zone B which proves that Ni and Mo have a strong solid solution ability. The content of Mo in Zone C further decreases, while the contents of Cr, Fe and Ni increase. There is no Mo element in Zone D.

**Figure 9:**
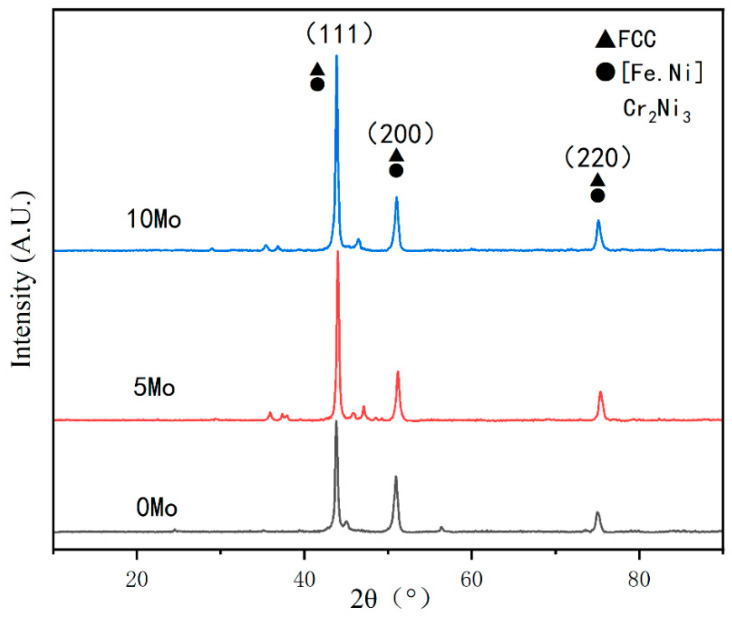
XRD results of top cladding layers with different contents of Mo.

Figure 9 shows XRD results of top covering layers with different contents of Mo. The diffraction peak position of covering layers does not change with the increase of Mo content, and the diffraction intensity is obvious, which proves that the crystallization at the top of the covering layers is excellent. The diffraction peak intensity of the crystal plane (111) is significantly higher than the two other peaks, which proves that the crystal plane (111) is the main grain growth orientation of covering layers. Moreover, the diffraction peak intensity of the crystal plane (111) increases obviously with the increase of Mo content, which shows that the addition of Mo promotes the growth of grains at the (111) crystal plane. 

The XRD analysis software was used for Figure 9, and the results show that there are austenite phases such as Ni_2_._9_Cr_0_._7_Fe_0_._36_ and Fe_0_._64_Ni_0_._36_ besides [Fe.Ni] solid solution at the top of the cladding layers. In addition, the intensity of the crystal plane (111) gradually increases with the increase of Mo content. The reason may be that there are [Ni.Mo] solid solution or compounds forming at the crystal plane (111). The peaks with very small diffraction intensity may stand for the austenite phases, such as CrFe_2_._32_MoNi.

### 3.2. Mechanical Property

**Figure 10:**
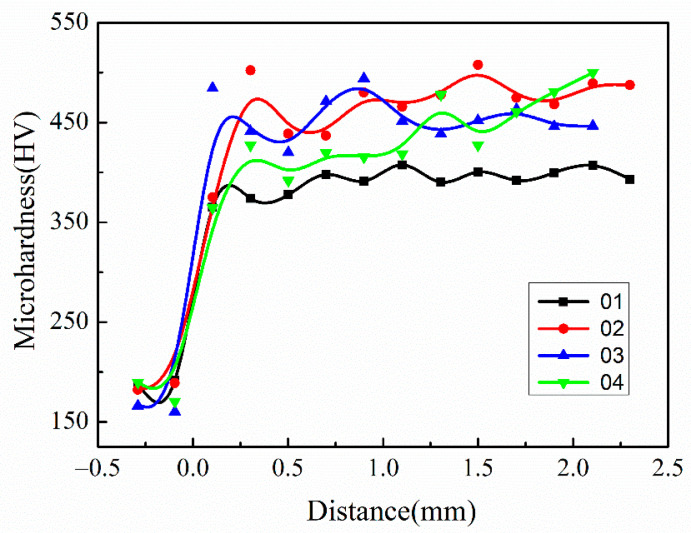
Comparison of microhardness of cladding layers with different contents of Mo.

The hardness of covering layers with different contents of Mo was tested, and the results are shown in Figure 10 and Table 7. The addition of Mo improves the hardness of covering layers significantly. The reason is that Mo atoms easily form solid solutions with Ni atoms in the molten pool owing to much larger radiuses than that of Ni, and the hardness increases due to the lattice distortion. In addition, the solidification time of the molten pool becomes longer because of the high freezing point of Mo, which facilitates the diffusion of elements and the homogenization of the structure and improves the hardness of covering layers. The average hardness of Sample 2 is the highest. The hardness of Sample 2 increases by 19% compared with Ni-Cr covering layers and about 159% compared with the matrix. However, the hardness decreases when the content of Mo continues increasing. The reason is that the number of unmelted Mo particles increases with more addition of Mo, which promotes generation of defects around particles and microstructure coarsening.

**Figure 11:**
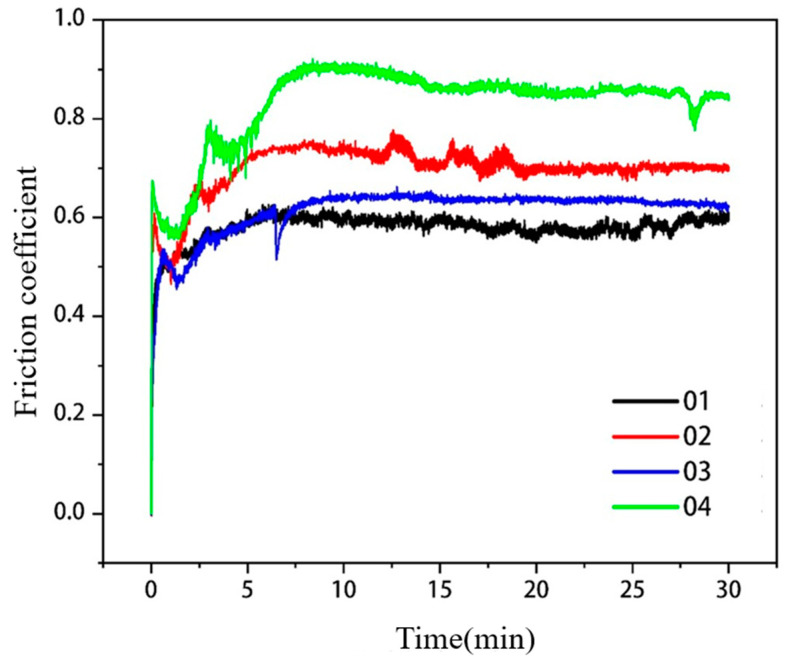
Comparison of friction coefficients of cladding layers with different contents of Mo.

The comparison of friction coefficients of covering layers with different contents of Mo are visualized in Figure 11. The average friction coefficients of the four covering layers are 0.5779, 0.6989, 0.6130 and 0.8310, respectively. The friction coefficient of the covering layer without Mo is the lowest. To accurately characterize the friction resistance of covering layers, the volume loss of covering layers was calculated, and the results are shown in Table 8. The volume loss of Sample 2 is the smallest, which is 59.8% of the loss of Sample 1. The volume loss of Sample 4 is the largest and even higher than that of the NiCr covering layer, which indicates that the friction resistance of covering layers is adversely affected when the content of Mo exceeds 15 wt.%. The microstructure of different covering layers after the friction test were analyzed to explore the reasons. The friction SEM images of covering layers are shown in Figure 11, and EDS analysis results of the friction zones are shown in Table 9.

**Figure 12:**
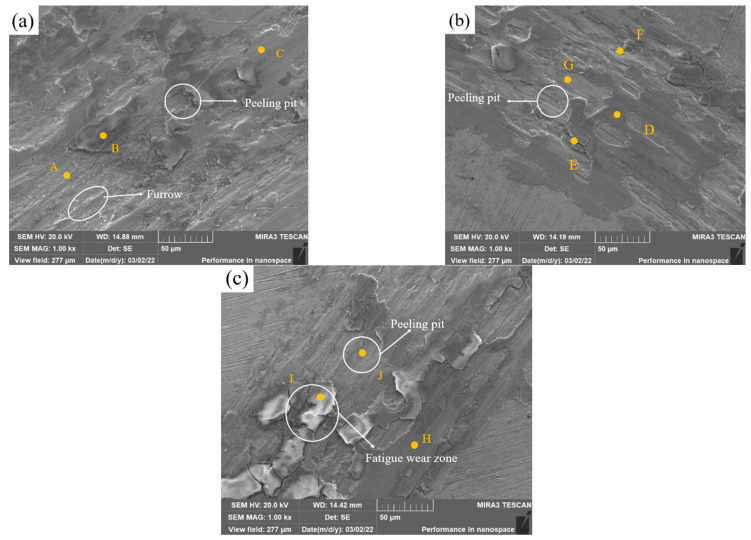
Friction SEM images of cladding layers with different contents of Mo. (**a**) 0 wt.% Mo. (**b**) 5 wt.% Mo. (**c**) 10 wt.%Mo.

Figure 12a shows the SEM image of Sample 1 after wear, revealing debris, deep furrows and a few peeling pits on the surface. The shear force causes the plastic deformation of the covering layer, and the abrasive wear is the main wear mode. According to Table 9, the phase of Zone A with spalling pits is rich with Fe and Ni that has not been oxidized. Zone B is a severely oxidized area, and the NiO, Fe_2_O_3_ and other oxide layers play a lubricating role. It is speculated that the original oxide protective layer above Zone A is spalled under the cyclic stress. Zone C is slightly oxidized, which proves that Fe, Ni and other elements form oxide layers on the surface of the covering layer in the friction test.

In Figure 12b, the number of furrows and peeling pits reduces obviously. In addition, a large area of oxide layers can be observed on the surface. The layer in Zone E has the highest content of O element where the layer was oxidized most seriously, and there are some cracks around the zone. The image of zones F and G shows the microstructure after the exfoliation of oxide layers, and the O content is almost 0 in the two zones. Zone D is almost intact. It is speculated that there are three stages in the friction process of the covering layer. First, the groove temperature rises rapidly, and Ni, Cr, Mo and other elements form oxide films on the surface, as in Zone D. Then, fatigue wear and gradually cracks appear on the oxide layers, as in Zone E, as the degree of oxidation increases. Last, the oxide layers slowly peel off, as in zones F and D. On the one hand, Mo and Ni form a solid solution, which enhances the hardness of the covering layer and improves its friction resistance. On the other hand, oxide films formed by Mo, such as MoO_2_, MoO_3_ and NiMoO_4_, play a better lubrication role in the friction process. 

In Figure 12c, the layer of Sample 3 is severely worn. The O content of Zone I is very large, which indicates that covering layers are oxidized seriously. Moreover, there are many cracks around the oxide layers which are almost peeling off. It is speculated that the main wear mode of the covering layers in Zone I is fatigue wear. The content of Mo in Zone J is higher than that in zones H and I and the oxide layers in Zone J have already fallen off to reveal the covering layer, which indicates that excess Mo is unfavorable to the friction resistance of covering layers.

### 3.3. Corrosion Resistance Test

**Figure 13:**
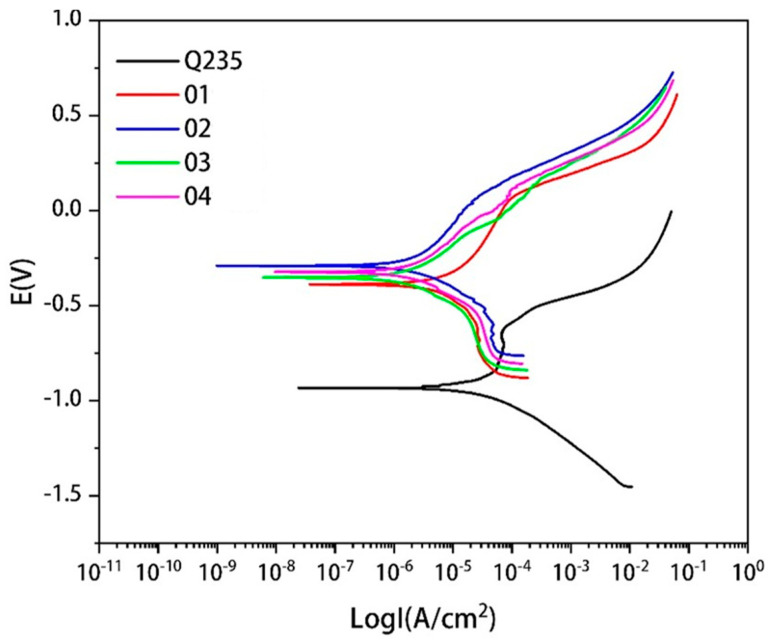
Polarization curves of matrix and cladding layers with different contents of Mo.

The polarization curves of the Q235 steel and covering layers with different contents of Mo in 3.5%NaCl solution are presented in Figure 13. There are active dissolution zones and over-passivation zones in the polarization curves of the Q235 steel and covering layers. In the active zone, the corrosion current density increases with the increase of the electrode potential. In this zone, many bubbles emerge from the surface of the samples and the metal becomes ionic. There are obvious passivation zones in the curves of Q235 and Sample 1. With the increase of the electrode potential, the variation of the corrosion current density is very little and stable passivation films are generated on the metal surface to protect the metal. However, the passivation zones in covering layers curve can not be found easily with the increase of Mo content. The reason may be that the addition of Mo causes the segregation of elements and the deposition of unmelted Mo particles and affects the production of passivation films. The addition of Mo reduces the pitting corrosion potential of covering layers, which indicates that Mo is beneficial in improving the pitting resistance of covering layers in NaCl solution.

**Figure 14:**
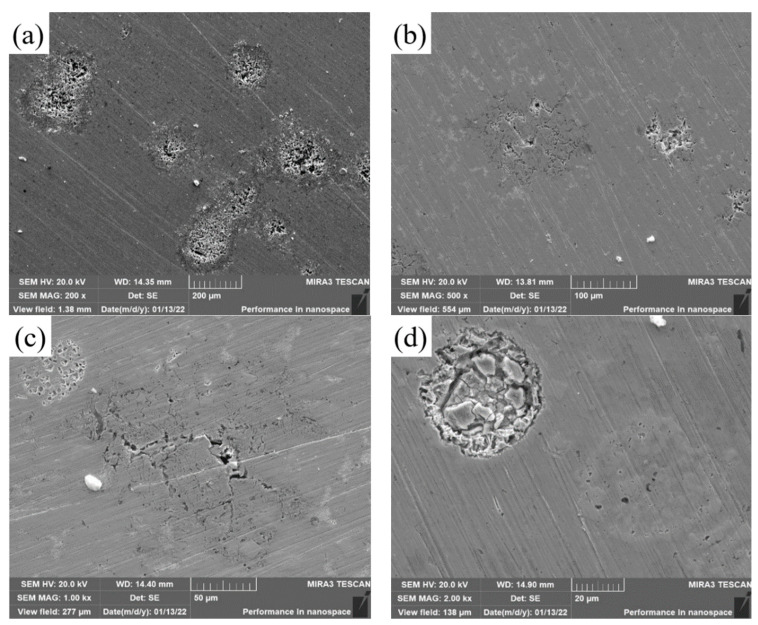
SEM images of cladding layers with different contents of Mo after corrosion. (**a**) 0 wt.% Mo. (**b**) 5 wt.% Mo. (**c**) 10 wt.% Mo. (**d**) 15 wt.% Mo.

Figure 14 shows SEM images of covering layers with different contents of Mo after corrosion. The surface of Sample 1 without Mo has manypitting pits and pores. The pitting pits of samples 2 and 3 reduce significantly, but there are small cracks in the corrosion zone. In Figure 14d, there is a large circular corrosion pit in Sample 4 where there are many lumps and pits.

**Figure 15:**
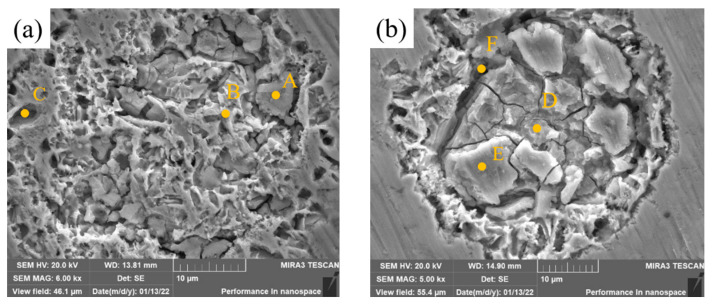
High-magnification SEM images of corrosion zones of cladding layers with different contents of Mo. (**a**) Sample 2. (**b**) Sample 4.

The high-magnification SEM images of the corrosion zones of samples 2 and 4 are presented in Figure 15, and the EDS analysis results are shown in Table 10. The layer in Zone A is rich in Mo and O, and oxide films of Mo generated during the corrosion process protect the metal. The layer in Zone B has high contents of Ni, Mo and O. It is possible that solid solutions of Ni and Mo and oxygen form composite oxides which are more resistant to Cl^−^ than NiO and the microstructure is retained. The contents of Ni and Mo in Zone C are similar to those in Zone B and the content of O is almost zero, which indicates that there is no oxide film here. The original Ni-rich structure in Zone C has been corroded, and the original structure with stronger corrosion resistance is exposed below the pore. The contents of Mo and O in Zone D and E are very high, and the contents of Cr and Fe are almost 0, which indicates that the structure of this zone is mainly unmelted Mo particles and that oxide films do indeed form during the corrosion process. However, the oxide films are spalling, and there are many cracks in the spalling zone. Elemental analysis of Crack F shows that the component segregation is serious and pores easily form, which is not conducive to the formation of stable passivation films. The layer is easily penetrated by Cl^−^. This is the reason why the corrosion resistance of samples 3 and 4 is lower than that of Sample 2.

## 4. Conclusions

(1) Ni-Cr-Mo plasma covering layers have an excellent performance and a good metallurgical combination with the Q235 matrix. With the increase of Mo content, the structure of the austenite-precipitated phase changes from dots and needles to granules uniformly distributed on the matrix.
(2) The hardness of covering layers first increases and then decreases with the increase of Mo content. The covering layer with 5 wt.% content of Mo has the highest hardness, of 466 HV, which is 159% higher than that of the matrix.
(3) Sample 2 with 5 wt.% content of Mo has the best wear resistance in which the number of furrows and peeling pits is the least. With the increase of Mo content, the main wear mode of covering layers changes from abrasive wear to fatigue wear.
(4) Sample 2 with 5 wt.% content of Mo has the best corrosion resistance. Mo is beneficial in improve the pitting resistance of covering layers and the number of pitting pits of the layer with 5% content of Mo is the least.

## Figures and Tables

**Table 1:** Chemical Composition of Q235 Substrate (wt.%).

C	Mn	Si	S	P	Fe
≤0.22%	≤1.4%	≤0.35%	≤0.050%	≤0.045%	≥97.935

**Table 2:** Cladding material composition (wt.%).

Sample Number	Ni	Cr	Mo
1	85	15	0
2	80	15	5
3	75	15	10
4	70	15	15

**Table 3:** Parameters of cladding process.

Ion Gas Flow Rate	Protective Gas Flow Rate	Powder Gas Flow Rate	Powder Feeding Rate	Cladding Current
3 L/min	9 L/min	9 L/min	40–60 g/min	170 A

**Table 4:** EDS analysis results at the bottom of the cladding layer (wt.%).

Zone	Ni	Cr	Fe
A	60.23	11.70	28.07
B	20.70	59.43	19.87
C	52.81	22.62	24.57

**Table 5:** EDS analysis results of cladding layer (wt.%).

Sample	Zone	Ni	Cr	Fe	Mo	C
2	A	11.53	43.65	8.37	33.30	3.15
	B	49.49	23.31	17.32	9.88	0
	C	80.21	6.71	9.28	3.80	0
	D	72.35	9.92	17.73	0	0
3	E	10.29	26.62	4.65	56.10	2.34
	F	37.22	26.44	9.29	25.94	1.11
	G	53.15	19.56	11.92	15.37	0
	H	66.32	14.81	12.62	6.25	0

**Table 6:** EDS analysis results of unmelted Mo particles (wt.%).

Zone	Ni	Cr	Fe	Mo
A	0	0	0	100
B	23.59	7.27	4.96	64.18
C	50.26	14.89	12.25	22.65
D	75.64	10.54	13.82	0

**Table 7:** Average hardness of cladding layers with different contents of Mo (HV).

Sample	1	2	3	4
Average hardness HV	391.4	466.5	455.3	435.1

**Table 8:** Friction coefficient and volume loss of cladding layers with different contents of MO.

Sample	Friction Coefficient	Groove Width (mm)	Volume Loss (mm^3^)	Volume Loss Rate (%)
1	0.5779	0.285	1.84 × 10^−2^	0.00164
2	0.6989	0.225	1.10 × 10^−2^	0.00098
3	0.6130	0.262	1.13 × 10^−2^	0.00100
4	0.8310	0.337	2.26 × 10^−2^	0.00201

**Table 9:** EDS analysis results of the friction zone of the cladding layer (wt.%).

Sample	Zone	Ni	Cr	Fe	Mo	O
1	A	38.24	6.97	54.79	0	0
	B	22.91	5.03	31.73	0	40.33
	C	38.08	6.65	48.57	0	6.70
2	D	50.12	9.12	22.59	2.68	15.49
	E	34.74	8.57	17.42	2.55	36.72
	F	54.22	13.53	26.31	3.30	2.64
	G	60.02	10.25	27.23	2.50	0
3	H	36.95	8.97	32.08	5.04	16.60
	I	26.77	6.43	23.73	4.37	38.71
	J	42.71	9.70	37.26	7.17	3.16

**Table 10:** EDS analysis results of corrosion area of cladding layer (wt.%).

Sample	Zone	Ni	Cr	Fe	Mo	O
2	A	5.74	5.55	3.34	55.94	29.43
	B	41.00	6.49	11.25	28.75	12.52
	C	41.47	14.53	16.26	26.28	1.46
4	D	2.80	0	0	67.52	29.68
	E	4.69	4.26	0	60.53	30.52
	F	40.10	12.32	7.14	37.85	2.58
